# Tumor Necrosis Factor-Alpha’s Role in the Pathophysiology of Colon Cancer

**DOI:** 10.3390/diseases13060185

**Published:** 2025-06-15

**Authors:** Saleha Khan, Yara Aldawood, Ayesha Hanin Shaikh, Aleena Zobairi, Urwa Nabilah, H. M. Alqahtani, Mansoor-Ali Vaali-Mohammed

**Affiliations:** 1 General Medicine Practice Program, Batterjee Medical College, Jeddah 21442, Saudi Arabia; yaraoad2004@gmail.com (Y.A.); ayesh03shk@gmail.com (A.H.S.); zobairi.aleena@gmail.com (A.Z.); 2 Shadan Institute of Medical Sciences, Affiliated with Kaloji Narayana Rao University of Health Sciences (KNRUHS), Telangana 500086, India; urwanabilah@gmail.com; 3 Stem Cell Unit, Department of Anatomy, College of Medicine, King Saud University, Riyadh 11472, Saudi Arabia; alqhamad@ksu.edu.sa; 4 Colorectal Research, College of Medicine, King Saud University, Riyadh 11472, Saudi Arabia

**Keywords:** colon cancer, tumor necrosis factor-alpha, inflammatory pathway, tumor, oxidative stress, DNA damage, therapeutic targeting

## Abstract

Colon cancer remains a significant global health challenge, with inflammatory pathways such as TNF-α playing a central role in its progression. TNF-α, a key proinflammatory cytokine, is implicated in various stages of colon cancer development, including inflammation, tumor growth, and metastasis. This review provides a comprehensive overview of the molecular mechanisms through which TNF-α contributes to colon cancer progression, with a focus on its interaction with signaling pathways like NF-κB and the Wnt/β-catenin in humans. TNF-α’s involvement in promoting tumorigenesis and its complex role in the tumor microenvironment highlight its potential as both a therapeutic target and a challenge for effective treatment. This review explores the potential of anti-TNF-α therapies and the emerging role of combination strategies with immune checkpoint inhibitors. Despite promising preclinical findings, clinical application faces challenges due to the dual role of TNF-α in both promoting and inhibiting tumor progression. Future research should aim to overcome resistance mechanisms, develop personalized therapeutic strategies, and balance the effects of TNF-α in cancer therapy.

## 1. Introduction

Colon cancer is the third most frequent cancer globally, and is more common in developed nations, especially in North America, Europe, and Australia. Rates are rising in Asia and Eastern Europe, particularly among older age groups, whereas they are stabilizing or declining in high-income regions [1].

Treatment options for colon cancer include radiation therapy, chemotherapy, targeted therapy, cryosurgery, and surgery. Surgery is the main treatment for colon cancer, and while it cures about 50% of patients, recurrence is a common problem. Chemotherapy is a crucial treatment approach, but it has severe side effects such hematological issues, gastrointestinal toxicity, and systemic toxicity from non-targeted drug distribution, which also leads to drug resistance. Although outcomes are suboptimal, advancements in drug delivery techniques and molecular biomarker identification are intended to increase treatment selectivity and decrease side effects [2]. Rather than genetic mutations, environmental factors have been implicated in most colon cancer cases. Certain intestinal pathogens and commensals, environmental and food-borne mutagens, and persistent intestinal inflammation—which occur before tumor development—are risk factors. An important factor in the onset and spread of colon cancer is inflammation. In diseases such as inflammatory bowel disease (IBD), chronic intestinal inflammation promotes DNA damage, epigenetic modifications, and the generation of reactive oxygen and nitrogen species, all of which can result in tumor suppressor gene mutations. By secreting proinflammatory cytokines and chemokines, this inflammatory environment also promotes tumor growth, metastasis, and progression. One of the main proinflammatory cytokines in this process is tumor necrosis factor-alpha (TNF-α). In colon cancer, TNF-α stimulates transcription factors like nuclear factor kappa-light-chain-enhancer of activated B cells (NF-κB) and signal transducer and activator of transcription 3 (STAT3), which promote angiogenesis, cell proliferation, survival, and immune evasion. Both sporadic and colitis-associated colorectal cancers have been found to have elevated TNF-α levels, which makes them an important target for therapeutic intervention [3].

This review provides comprehensive overview of the literature analyzing TNF-α’s complex function in colon cancer pathogenesis by bridging the gaps between inflammation, cancer progression, and therapeutic targeting.

The main objective of this review is to examine how TNF-α affects the pathophysiology of colon cancer, with a strong focus on emphasizing its regulation of inflammation, tumor growth, and immune response. To improve treatment outcomes for individuals with colon cancer, the literature review combines previous research into coherent models of TNF-α mediated cancer progression in humans and restructure that suggests possible therapeutic interventions that may direct future studies and clinical approaches.

## 2. Role of Tumor Necrosis Factor-Alpha in Colon Cancer Pathophysiology

### 2.1. Structure and Function of TNF-α

TNF-α is well known for its capacity to destroy tumors and was known as cachexin due to this function; however, this cytokine has a complex role in the tumor microenvironment of colorectal carcinoma (CRC). The family of TNF proteins consists of membrane proteins and secreted cytokines that bind to the cell surface receptors. As stated earlier, activated macrophages, T cells, various immune cells including natural killer cells, and even tumor cells produce TNF-α [4]. At the molecular level, the TNF-α is synthesized as a type 2 transmembrane precursor protein (tmTNF) weighing 26 kilodaltons (kDa). The TNF-α converting enzyme (TACE), a matrix metalloproteinase, then cleaves the tmTNF, releasing a soluble molecule (sTNF) that weighs 17 kDa. The tmTNF and sTNF unify into groups of three, forming a homotrimer to activate their cell function. The TNF gene, located on chromosome 6, codes for the homotrimer TNF-α protein consisting of 157 amino acids. Two distinct cell surface receptors, TNF receptor 1 (TNFR1) and TNF receptor 2 (TNFR2), mediate the biological actions and signaling pathways of TNF-α. Among these two receptors, TNFR1 can initiate most functions of TNF-α. It is heavily expressed on almost all cell types, whereas TNFR2 is mainly expressed by immune cells [5]. The TNFR1 is widely studied for its double role; it helps in facilitating cell death or apoptotic signals due to the presence of the death domain (DD), but can also induce cell proliferation and survival signals. The death domain is the cytoplasmic domain of the type 1 receptor that aids in initiating signaling pathways, as the TNFR lacks its own enzyme [6]. The primary signaling pathway in the case of TNFR1 is the anti-apoptotic pathway, where nuclear factor kappa-B (NF-κB) is activated for cell survival. TNFR2 has a pro-survival function, where the signaling pathway is activated via NF-κB [5].

### 2.2. Tumor Necrosis Factor Alpha (TNF-α) and Colorectal Carcinoma

One of the most common malignancies of the gastrointestinal tract is colorectal carcinoma, and it has also become one of the leading causes of cancer-related deaths worldwide [7]. Tumor cells release inflammatory cytokines to induce inflammation that significantly contributes to the progression of malignancy. TNF-α has various biological actions like inflammation, apoptosis, cell proliferation, and differentiation. However, in the TME of colorectal cancer, elevated levels of TNF-α have been recognized to play a role in tumor progression and metastasis. The tumor surrounding cells produce proinflammatory cytokines as well as various growth factors that accelerate the process of cell transition. The epithelial cells of a tumor acquire mesenchymal-like properties, and this process is called epithelial mesenchymal transition (EMT). These cells gain invasive and migratory properties and play an important role in the progression and metastasis of CRC. To evaluate the proliferation and migratory potential of cells treated with TNF-α, a wound healing assay was performed. In both cell lines, TNF-α treatment rapidly closed the wound compared with the controls (*p* < 0.0001), and this result demonstrated the role of TNF-α induced EMT in colorectal cancer cases [8]. Another study conducted by Wang et al. revealed that TNF-α induced EMT in highly aggressive human colorectal carcinoma cell line HCT116 accelerated the cancer invasion and metastasis [9]. A study investigating the relationship of serum levels of TNF-α with survival and progression of cancer in CRC patients produced significant results. The study included 119 CRC cases and 177 controls, recording the highest levels of TNF-α in terminal stage CRC (42.7 ± 12.5 pg/mL). Additionally, patients with low serum levels of TNF-α had significantly higher median survival rates compared to those with high levels of TNF-α [10].

Early stages of CRC exhibit low serum levels of TNFα, whereas the advanced stages show elevated levels. High levels of TNF-α also influence the activation of nuclear factor κB (NF-κB) and activator protein 1 (AP-1) through pro-oncogenic signaling pathways that help in cell survival and proliferation in CRC. A recent study evaluating the effect of TNFα on HT-29 colorectal cancer cells observed that TNFα secreted high levels of pro-tumorigenic cytokines IL-8 and IL-6, that aided tumor progression [7]. A downstream transcription factor of the cytokine IL-6 is STAT3, which plays an indispensable role in the development of various cancers, including CRC. TNFα plays an important role in increasing the phosphorylation and expression of STAT3 and its target genes related to cell survival and metastasis of colorectal cancer [11].

Tumor necrosis factor-α-induced protein 8 (TNFAIP8/TIPE) is a family of proteins whose expression is regulated by TNFα. This family has many types of proteins, including TNFAIP8-like 1 (TIPE1), TNFAIP8-like 2 (TIPE2), and TNFAIP8-like 3 (TIPE3) proteins. It is to be noted that overexpression of TNFAIP8 is associated with the pathogenesis of various cancers including CRC [12]. A 2014 study was conducted to better understand the mechanism of TIPE2 via caspase 8 in colon cancer patients, and the study discovered that TIPE2 was dominantly expressed in colon cancer tissues and its expression was linked to lymph node distant metastasis and the Dukes stage of CRC [13]. Multiple other studies have recorded a relationship between TNF-α and colon cancer, as shown in Table 1.

### 2.3. Tumor Necrosis Factor Alpha (TNF-α) and Immune Response

Upon recognition of tumor cells, the body’s immune response fuels inflammatory changes that contribute to carcinogenic processes. This inflammation feeds tumor growth by releasing bioactive molecules like growth factors, cytokines, and chemokines. A weakened immune response aids in the progression of malignant neoplasm. In the case of CRC, tumor cells acquire characteristics that help the tumor in evading immunological surveillance [14].

**Figure 1:**
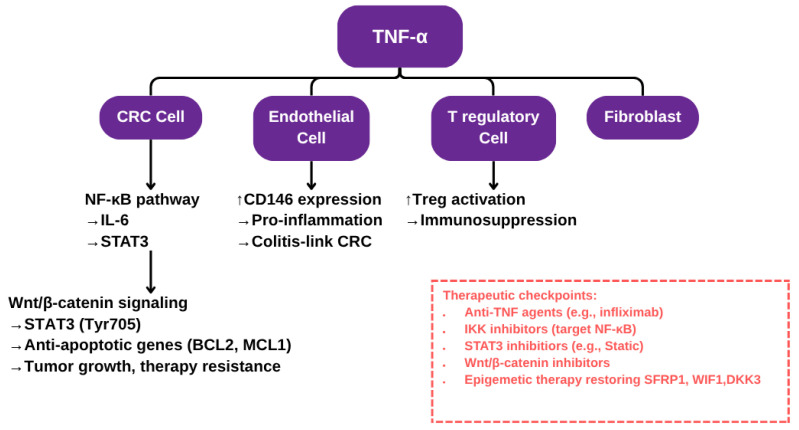
Schematic representation of TNF-α signaling in the colorectal cancer microenvironment. This diagram illustrates the multifaceted role of TNF-α in promoting colorectal cancer (CRC) progression through interactions with various cell types within the tumor microenvironment. In CRC cells, TNF-α activates the NF-κB signaling cascade, inducing IL-6 production which further enhances Wnt/β-catenin signaling and upregulates anti-apoptotic genes, contributing to tumor growth and resistance to therapy. In endothelial cells, TNF-α upregulates CD146 expression and promotes a pro-inflammatory phenotype, linking chronic inflammation to CRC development, particularly in colitis-associated cases. In regulatory T cells (Tregs), TNF-α fosters Treg activation, reinforcing an immunosuppressive environment that supports tumor immune evasion. The red box highlights key therapeutic checkpoints, including anti-TNF agents and epigenetic therapies aimed at restoring tumor suppressor genes.

TNFα has context-dependent effects on human colon cancer cells. It is predominantly pro-tumorigenic in vitro, promoting cell proliferation, migration, invasion, and resistance to apoptosis through activation of NF-κB, STAT3, ERK1/2, and EMT pathways [20,24] (see Figure 1 for a schematic representation of TNF-α signaling in the colorectal cancer microenvironment). TNFα also contributes to DNA damage via the JNK pathway and enhances stromal COX-2 signaling, which supports epithelial tumor progression [25,26]. However, under specific molecular conditions, such as inhibition of anti-apoptotic proteins or S-nitrosylation of key survival regulators, TNF-α can induce apoptosis and suppress tumor traits [27]. These findings from human studies underline the dual and context-sensitive role of TNFα in colorectal cancer pathogenesis, as shown in Table 2.

#### 2.3.1. Pro-Inflammatory Role and Immune Activation

In the early stages of CRC, TNF-α generates an inflammatory response in which immune cells such as neutrophils and macrophages are recruited to tumor site. The prominent subtype of neutrophils, known as N1, is initially released and transforms into NF2 as the cancer progresses [29]. One of the key signaling pathways responsible for the transcription of pro-inflammatory genes is the NF-κB pathway that is activated via the TNF-α receptors (TNFR1 and TNFR2). Natural killer (NK) cells are a primary defense against tumor cells. These cells include both types of receptors, activating and inhibitory receptors. Laboratory investigations in CRC patients have shown a decrease in type 1 NK cells, whilst there has been an increase in type 2 NK cells in the early stages of cancer. The member D of group 2 (NKG2D) acts as an activating receptor, while the member A (NKG2A) is an inhibitory receptor. The interaction between the ligands of NKG2D (NKG2DL) and NKG2D itself influences the survival, expansion and cytotoxic activity of NK cells, that triggers a strong immune response against colorectal cancer cells [30].

TNF-α activates the T cells and aids in maturating the antigen presenting dendritic cells. Certain inflammatory cytokines studied earlier, like IL-6, which are produced by TNF-α, also amplify the immune response in TME during early stages [6,7].

#### 2.3.2. Immune Evasion

Immune evasion is a strategic mechanism that allows cancerous cells to go unnoticed by the immune system. In CRC patients, this mechanism is mediated by the dual role of TNF-α. The regulatory T cells (Tregs) are expressed by the action of TNF-α, and they undermine the function of the effector T cells. Tregs contribute by activating a complex network of signaling pathways, leading to immunosuppression and tumor progression in CRC. The two most important pathways activated by the Tregs are PI3K/Akt/mTOR and the NF-κB pathway, both of which affect the effector T cells, as those cells are the pathways activation site. TNF-α plays a key role in enhancing the Treg cell stability and its suppressive function. The effector T cells are responsible for fighting off tumor cells; however, due to the activation of these pathways, the prime ability of the effector T cells, to produce inflammatory cytokines and eliminate the tumor cells, is greatly reduced. Moreover, the Tregs helps in activation of the STAT3 pathway by acting on tumor cells to promote immune evasion and tumor growth. A recent study confirmed that tumor progression is halted due to the depletion or blockade of TNFR2. This blockade also reduces CCR8+ Treg invasion and enhances the effectiveness of anti-PD1 therapy [23].

Another factor that helps tumor cells to evade the immune system is the immune checkpoint inhibitors expressed by the tumor cells in response to TNF-α. The increased expression of immune checkpoint inhibitor programmed death-ligand 1 (PD-L1) downregulates the T-cell responses, helping the tumor cells to escape destruction by the immune system [13].

## 3. Mechanistic Insights of Pathways

### 3.1. NF-κB Pathway Activation

The NF-κB signaling pathway plays a central role in regulating inflammation, immune responses, and cancer development, especially in CRC. Its dysregulation is a key driver in the onset and progression of chronic inflammatory diseases such as inflammatory bowel disease (IBD) and colitis-associated cancer (CAC). NF-κB, a family of transcription factors, is crucial for maintaining intestinal homeostasis, preserving epithelial integrity, and modulating interactions between the gut epithelium and the microbiota. In IBD, NF-κB is activated in macrophages and epithelial cells within the inflamed mucosa, contributing to both inflammatory signaling and epithelial barrier dysfunction [31,32,33,34].

NF-κB is activated by a variety of stimuli, including pro-inflammatory cytokines such as tumor necrosis factor-alpha (TNF-α). TNF-α binds to its receptor TNFR1, initiating a signaling cascade that culminates in the nuclear translocation of NF-κB. Once in the nucleus, NF-κB activates the transcription of genes involved in inflammation, immune responses, and cell survival. This interaction is vital in modulating both the immune response and the resolution of inflammation. Moreover, TNF-α can trigger cell death mechanisms such as apoptosis and necroptosis, processes that are tightly regulated by NF-κB activity. For example, NF-κB can inhibit apoptosis by inducing anti-apoptotic proteins like c-FLIP, while its absence may lead to enhanced cell death. This dual role underscores the importance of the TNF-α–NF-κB axis in balancing immune responses, tissue repair, and cellular fate, all of which are central to both immune defense and disease pathogenesis, including cancer [35].

In colon cancer, NF-κB is activated through both the canonical and non-canonical signaling pathways. The canonical pathway—primarily stimulated by TNF-α—begins when TNF-α binds to TNFR, leading to the recruitment of the IκB kinase (IKK) complex, which is composed of IKKα, IKKβ, and NEMO, via adaptor proteins such as TRAF2 and RIP1. This interaction promotes K63-linked ubiquitination of RIP1, mediated by E3 ligases cIAP1 and cIAP2, forming a scaffold for IKK activation. The IKK complex is then phosphorylated by upstream kinases like MEKK3 and TAK1. Activated IKK phosphorylates IκB proteins, targeting them for polyubiquitination and subsequent proteasomal degradation (see Figure 1). This degradation frees NF-κB dimers (e.g., p65/p50), allowing them to translocate into the nucleus and initiate transcription of pro-inflammatory and survival-related genes. Evidence from CAC models suggests that cell-specific NF-κB activity plays distinct roles in tumor biology. For instance, deletion of IKKβ in intestinal epithelial cells reduces tumor incidence without significantly affecting tumor size, indicating a limited role in tumor progression. Conversely, deletion of IKKβ in myeloid cells significantly decreases tumor size, highlighting the tumor-promoting role of macrophage-derived NF-κB signaling. Additionally, conditional ablation of NEMO in intestinal epithelial cells causes severe colitis and barrier dysfunction, further emphasizing the essential role of NF-κB in epithelial integrity and inflammatory regulation.

**Figure 2:**
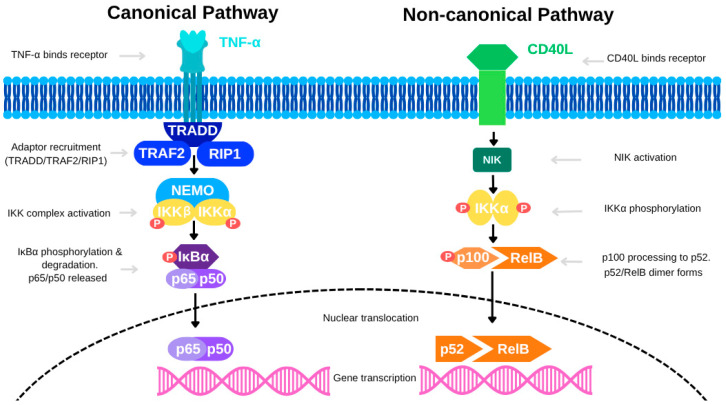
The canonical and non-canonical NF-κB signaling pathways.

The non-canonical NF-κB pathway also contributes to colon cancer, particularly in regulating immune functions and cell survival. Activated by receptors such as BAFFR, CD40, and LTβR, this pathway leads to the stabilization of NF-κB-inducing kinase (NIK), which drives the processing of the precursor p100 into p52. The resulting p52/RelB complex translocates into the nucleus to control gene expression (see Figure 2). Although slower and more sustained than the canonical pathway, the non-canonical pathway is essential for maintaining long-term immune responses and tissue remodeling within the colon. Redox signaling further modulates NF-κB activation. Reactive oxygen species (ROS), generated by stimuli like TNF-α and IL-1, can oxidize cysteine residues on NF-κB subunits, enhancing their DNA-binding affinity and transcriptional activity. Similarly, lipid signaling molecules such as ceramide, produced from diacylglycerol (DAG) through sphingomyelinase activation, facilitate NF-κB signaling by modulating relevant kinases and phosphatases.

Given NF-κB’s multifaceted role in inflammation and tumorigenesis, it represents a promising therapeutic target for colon cancer treatment. Preclinical studies underscore the importance of TNF–NF-κB signaling in both tumor initiation and progression, as well as in maintenance of mucosal homeostasis. Therapeutic interventions aimed at key NF-κB components, such as IKKβ, NEMO, or TNF receptors, could potentially treat CAC and related inflammatory conditions [31,32,33,34]. However, targeting NF-κB in colorectal cancer is challenging because it is activated in multiple cell types within the tumor microenvironment (TME), including macrophages, epithelial cells, and stromal components. For instance, TNF-α-driven NF-κB activity in macrophages promotes tumor-associated inflammation and immunosuppression, whereas epithelial NF-κB is essential for barrier maintenance and epithelial function. Broad-spectrum NF-κB inhibition may therefore impair protective responses in the epithelium. To mitigate this risk, recent research has focused on cell-specific targeting strategies. Macrophage-directed delivery systems, such as nanoparticles conjugated with macrophage-specific ligands (e.g., anti-CD68 or mannose receptors), have shown promise in selectively delivering NF-κB inhibitors to immune cells while sparing epithelial tissue [35,36,37]. Additionally, exosome-based siRNA delivery systems and gene therapy vectors driven by macrophage-specific promoters (e.g., CSF1R or CD11b) offer precise tools to downregulate NF-κB signaling selectively in macrophages [38,39]. These targeted approaches may maximize therapeutic effectiveness while minimizing adverse effects on epithelial homeostasis.

The canonical pathway, activated by TNF-α, involves recruitment of adaptor proteins (TRADD, TRAF2, RIP1), leading to activation of the IKK complex (IKKα, IKKβ, NEMO). This triggers phosphorylation and degradation of IκBα, allowing the p65/p50 NF-κB dimer to translocate into the nucleus and drive gene transcription. In contrast, the non-canonical pathway is activated by CD40L, which induces NIK-mediated phosphorylation of IKKα. This promotes processing of p100 to p52 and formation of the p52/RelB dimer, which then translocates into the nucleus to regulate a distinct set of genes. [**Abbreviations**: TNF-α, Tumor Necrosis Factor alpha; CD40L, CD40 Ligand; TRADD, TNF Receptor-Associated Death Domain; TRAF2, TNF Receptor-Associated Factor 2; RIP1, Receptor-Interacting Protein 1; NEMO, NF-κB Essential Modulator; IKK, IκB Kinase; IKKα/IKKβ, Catalytic subunits of the IKK complex; IκBα, Inhibitor of NF-κB alpha; NIK, NF-κB Inducing Kinase; p65 (RelA), p50, p52, RelB, NF-κB family transcription factors; NF-κB, Nuclear Factor kappa-light-chain-enhancer of activated B cells].

### 3.2. TNF-α and Endothelial Cell Interactions

TNF-α and endothelial cell interactions promote CRC progression by inducing proinflammatory, procoagulant, and mesenchymal changes in both endothelial and cancer cells, primarily through NF-κB activation and related signaling pathways. In endothelial cells, TNF-α enhances TGF-β-induced endothelial-to-mesenchymal transition (EndMT), resulting in the generation of cancer-associated fibroblasts (CAFs) that support tumor growth via sustained Smad2/3 activation and upregulation of TGF-β signaling components [40]. Additionally, TNF-α stimulates epithelial-to-mesenchymal transition (EMT) in CRC cells, increasing their invasiveness and metastatic potential through NF-κB activation and upregulation of factors such as miR-105 [41,42,43]. TNF-α also triggers proinflammatory and procoagulant responses in endothelial cells, thereby promoting tumor cell migration, invasion, and metastasis via NF-κB signaling [44,45].

The key signaling pathways involved include the central role of NF-κB in mediating EMT, EndMT, and proinflammatory gene expression [41,42,44,45]; miR-105 in facilitating EMT and metastasis [42]; RIP1/WNT/β-catenin signaling in stabilizing β-catenin to promote EMT and metastasis [43]; and TNF-α-enriched extracellular vesicles that activate the NF-κB/LAMB3/AKT axis to support metastasis [45]. Collectively, TNF-α-modified endothelial cells create a tumor-supportive microenvironment via EndMT and EMT, both of which are critical for tumor invasion and metastasis, with NF-κB integrating inflammatory and oncogenic signals [41,42,43,44,45]. Thus, interactions between TNF-α and endothelial cells play a central role in CRC pathogenesis by driving inflammatory, mesenchymal, and pro-metastatic alterations in the tumor microenvironment.

### 3.3. Crosstalk with Wnt/β-catenin and STAT3 Signaling Pathways

CRC is often characterized by the dysregulation of the Wnt/β-catenin signaling pathway, a crucial cascade responsible for maintaining intestinal homeostasis. Under normal physiological conditions, the Wnt signaling pathway is tightly regulated to control cell proliferation, differentiation, and apoptosis. However, in CRC, epigenetic alterations frequently disrupt this regulation. DNA methylation of key Wnt antagonists such as the SFRP family (secreted frizzled related proteins), WIF1 (Wnt inhibitory factor 1), and DKK3 (Dickkopf related protein 3) has been well documented. These methylation events cause transcriptional silencing of essential Wnt inhibitors, thereby eliminating their ability to restrain the pathway. As a result, the Wnt/β-catenin signaling cascade becomes abnormally activated, contributing to uncontrolled cell proliferation, survival, and tumor progression, which are hallmark features of CRC.

Silencing of Wnt antagonists through epigenetic mechanisms results in the stabilization and nuclear accumulation of β-catenin, a central effector of the Wnt pathway. In the absence of Wnt inhibitors, β-catenin builds up in the cytoplasm, avoids degradation, and moves into the nucleus. Inside the nucleus, β-catenin binds to members of the T cell factor and lymphoid enhancer binding factor (TCF/LEF) family, forming a transcriptional complex that promotes the expression of genes responsible for proliferation, survival, and resistance to apoptosis. This aberrant activation of the Wnt/β-catenin pathway plays a vital role in CRC tumorigenesis, highlighting the potential of targeting epigenetic modifications to restore Wnt regulation and suppress tumor development.

One therapeutic approach to reversing the methylation-driven silencing of Wnt antagonists involves inhibiting DNA methyltransferases (DNMTs), the enzymes that add methyl groups to DNA. In CRC, DNMT inhibition has been shown to reduce the stem-like properties of cancer cells. This occurs through demethylation of genes that normally inhibit Wnt signaling, such as SFRP1, leading to reduced abnormal activation of the Wnt/β-catenin pathway. Specifically, SFRP1 methylation is essential for maintaining the cancer stem cell population in colorectal tumors. This suggests that reactivating SFRP1 expression via DNMT inhibition could impair the self-renewal capacity of CRC stem cells, limiting tumor progression and metastasis [37].

The interaction between Wnt/β-catenin signaling and TNF-alpha (tumor necrosis factor-alpha) is complex and involves intricate signaling crosstalk, especially in the context of inflammation and immune modulation. Wnt signaling, particularly through the β-catenin dependent pathway, controls essential cellular functions such as proliferation, differentiation, and apoptosis. TNF-alpha, on the other hand, is a major proinflammatory cytokine that activates the NF kappa B pathway to promote immune and inflammatory responses. Interestingly, β-catenin has been shown to interact with NF kappa B, with evidence suggesting that stabilized β-catenin can modulate NF kappa B activity, either enhancing or inhibiting its transcriptional function depending on the cellular environment. In inflammatory conditions such as inflammatory bowel disease (IBD), dysregulated Wnt/β-catenin signaling can intensify inflammation, whereas β-catenin activity in immune cells, particularly dendritic cells, contributes to balancing proinflammatory Th1 and Th17 responses with anti-inflammatory Treg cells. This signaling interplay offers a regulatory mechanism for immune tolerance and inflammation, where β-catenin functions as a molecular rheostat. Understanding this interaction could provide insights into novel therapeutic approaches for inflammatory and autoimmune diseases [37].

The canonical Wnt/β-catenin pathway is activated when Wnt ligands such as Wnt1, Wnt3a, or Wnt5a bind to membrane receptors including members of the Frizzled family and the co-receptor LRP5 or LRP6 (low density lipoprotein receptor related protein 5 or 6). This ligand-receptor engagement initiates a signaling cascade that stabilizes β- catenin. Under homeostatic conditions, β-catenin is regulated by a destruction complex composed of proteins such as APC (adenomatous polyposis coli), GSK3 beta (glycogen synthase kinase 3 beta), and CK1 (casein kinase 1), which facilitate its phosphorylation and subsequent degradation. However, when Wnt ligands are present, the destruction complex is inhibited, leading to β-catenin accumulation in the cytoplasm. Stabilized β-catenin translocates to the nucleus, where it binds to TCF and LEF transcription factors and initiates the transcription of genes that support cell cycle progression, cell survival, and differentiation.

**Figure 3:**
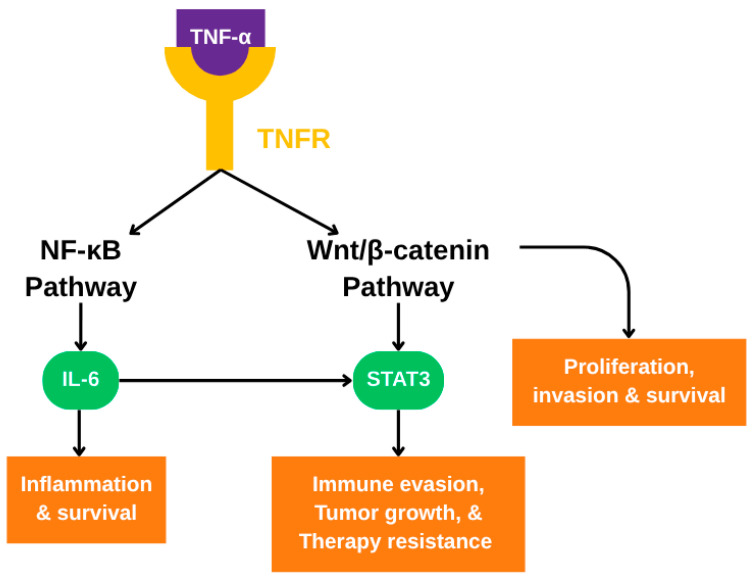
TNF-α-mediated signaling pathways involved in colorectal cancer progression. Upon binding to TNFR, TNF-α activates both the NF-κB and Wnt/β-catenin signaling pathways. NF-κB induces IL-6, which in turn activates STAT3. Concurrently, Wnt/β-catenin signaling also activates STAT3. Activated STAT3 promotes transcription of genes involved in immune evasion, tumor growth, and therapy resistance. The combined activity of these pathways contributes to inflammation, proliferation, invasion, and survival of colorectal cancer cells.

In CRC, the Wnt/β-catenin pathway not only drives tumor formation but also supports the survival of cancer cells under stress conditions such as oxidative stress, which is prevalent in the tumor microenvironment. Oxidative stress, driven by reactive oxygen species (ROS), damages cellular components and can induce apoptosis. However, Wnt signaling helps counteract this damage by activating survival pathways. One important downstream pathway involves the transcription factor STAT3 (signal transducer and activator of transcription 3). When Wnt signaling is active, it leads to the phosphorylation of STAT3 at tyrosine 705, a modification that activates STAT3 and facilitates its nuclear translocation. Inside the nucleus, STAT3 induces the expression of genes encoding survival proteins, including Bcl 2 and Mcl 1. This Wnt mediated activation of STAT3 is especially relevant in CRC cells, where it helps protect against oxidative stress and enhances cell viability, as illustrated in Figure 3. The essential role of STAT3 in this mechanism is supported by studies showing that STAT3 depletion reduces the protective effects of Wnt signaling. This indicates that STAT3 is a crucial mediator of Wnt driven survival in CRC. The Wnt–STAT3 axis thus represents a key pro-survival mechanism that allows CRC cells to persist under hostile conditions and maintain their tumorigenic potential. This further supports the idea that targeting components of this pathway may provide therapeutic value in CRC.

In conclusion, the Wnt/β-catenin signaling pathway is a central driver of CRC development, with its dysregulation often resulting from epigenetic silencing of pathway inhibitors. This leads to enhanced survival of cancer cells, especially under oxidative stress, through the activation of STAT3. Elucidating the molecular links between Wnt/β-catenin and STAT3 signaling offers valuable insight into CRC biology and potential therapeutic targets. Strategies that target both the epigenetic repression of Wnt antagonists and the Wnt–STAT3 signaling axis may offer promising approaches for CRC treatment and prevention [37,38,39,46,47,48].

## 4. TNF-α as a Therapeutic Target in Colon Cancer

TNF-α plays a critical and complex role in colon cancer progression. High TNF-α expression is associated with poor prognosis and is known to promote tumor growth and EMT, thereby enhancing tumor invasiveness [21,49,50,51]. However, TNF-α exhibits context-dependent effects: under certain conditions, it can also induce apoptosis in tumor cells [8,52,53,54]. This dual nature extends to its immunomodulatory roles. TNF-α can activate anti-tumor immune responses, yet it can also facilitate immune suppression within the tumor microenvironment, adding to the ongoing controversy surrounding its overall impact on colon cancer pathophysiology [54,55].

Therapeutically, targeting TNF-α has shown promise, particularly with monoclonal antibodies such as infliximab. These agents induce antibody-dependent and complement-dependent cytotoxicity, promoting tumor cell apoptosis [56]. When combined with chemotherapeutic agents like oxaliplatin or 5-fluorouracil, anti-TNF-α therapy has demonstrated synergistic effects that result in tumor regression in preclinical models [21,56]. Additionally, TNF-α inhibitors including infliximab, adalimumab, and etanercept have been shown to reduce inflammation and tumor growth in colorectal cancer models [49]. These inhibitors are especially relevant in cancers associated with chronic inflammation, such as those arising from ulcerative colitis.

Recent preclinical studies have expanded the therapeutic landscape by demonstrating that targeting TNF-α or its downstream pathways such as STAT3 and CSF-1 can significantly reduce tumor growth and metastatic potential [11,57]. Furthermore, TNFAIP6, a TNF-α-induced protein, is currently being explored as a drug target. Several potential therapeutic agents have been identified through pharmacological databases, although their efficacy in human clinical settings remains unconfirmed [58]. Importantly, TNF-α blockade is also being considered as a strategy to enhance immunotherapy, although its dual roles in immune activation and suppression complicate clinical translation [54].

Despite its promise, anti-TNF-α therapy is associated with significant adverse effects. These include increased susceptibility to opportunistic infections such as tuberculosis and pneumocystis, neurological complications like Guillain–Barré syndrome and multiple sclerosis, and autoimmune conditions including autoimmune hepatitis, psoriasis, and Crohn’s disease [59]. A retrospective study reported tuberculosis-related complications with all five TNF-α inhibitors examined, particularly in older individuals with weakened immunity [60]. Moreover, concerns regarding long-term safety persist, including the potential for secondary malignancy development, necessitating ongoing patient monitoring.

Novel therapeutic strategies are being explored to address these limitations. Selective inhibitors like takinib, which sensitize tumor cells to TNF-α-induced cell death, represent one such approach [60]. Additionally, the identification of small-molecule inhibitors that disrupt TNF-α activity offers another avenue for therapeutic development, as shown in Table 3 [61].

## 5. Limitations and Future Directions

This review focuses primarily on the role of TNF-alpha in colon cancer, potentially overlooking the contributions of other cytokines, signaling pathways, and molecular mechanisms that may also play important roles in disease progression. Furthermore, some of the information discussed is derived from preclinical studies, which may not fully represent clinical outcomes in human patients. The therapeutic potential of targeting TNF-alpha in colon cancer remains uncertain, largely due to the limited success of anti-TNF therapies in clinical trials for solid tumors, including colorectal cancer. So far, these therapies have shown efficacy mainly in treating inflammatory diseases rather than acting as effective anticancer agents, highlighting the difficulty of translating preclinical findings into clinical benefit. In addition, although TNF-alpha is explored as a therapeutic target in this review, the mechanisms of resistance that may arise during anti-TNF-alpha therapy are not comprehensively addressed, even though these mechanisms are critical for the development of more effective and durable treatment strategies.

Emerging approaches in precision medicine, such as targeted molecular therapies and personalized immunotherapy, offer new opportunities to overcome resistance and improve treatment outcomes. For example, combining immune checkpoint inhibitors with agents like CD40 agonists, or targeting alternative immune checkpoints, may enhance therapeutic efficacy. Furthermore, genetic profiling and advanced technologies such as circulating tumor cell detection may enable the development of more personalized and effective treatment approaches, especially for individuals at high risk [59,60,61]. Therapeutically targeting TNF-alpha is further complicated by its dual role as both a tumor suppressor and a tumor promoter, as well as its ability to promote metastasis through mechanisms such as microRNA 21 activation and TROP 2 overexpression. One of the major challenges in developing TNF-alpha based therapies is finding the right balance between these opposing effects [8,20,62].

This study clearly outlines the critical role that TNF-alpha plays in the pathophysiology of colon cancer, particularly in relation to inflammation, tumor progression, and immune regulation. It also provides valuable insights into how TNF-alpha contributes to colon cancer advancement by integrating current preclinical and clinical findings. In addition, it highlights the importance of continued research aimed at improving therapeutic strategies. Despite its limitations, TNF-alpha remains a compelling therapeutic target in colon cancer.

## 6. Conclusions

TNF-α plays a multifaceted role in the pathophysiology of colon cancer, acting as both a promoter and a suppressor of tumor progression. The cytokine’s involvement in inflammation, immune modulation, and the tumor microenvironment makes it a crucial factor in cancer development. While preclinical studies support the therapeutic targeting of TNF-α, clinical applications have shown mixed results, highlighting the complexity of balancing TNF-α’s dual roles. Future research should address key challenges in clinical translation, such as developing selective inhibitors that target pro-tumorigenic TNF-α signaling while maintaining anti-tumor effects, identifying predictive biomarkers to determine which patients may benefit from anti-TNF-α therapies, and optimizing combination strategies with immunotherapy or chemotherapy to prevent resistance mechanisms. Anti-TNF-α therapies, particularly monoclonal antibodies, have demonstrated promising effects in reducing inflammation and tumor progression, but the emergence of resistance mechanisms underscores the need for improved therapeutic strategies. Ultimately, a deeper understanding of TNF-α signaling and its interactions with other oncogenic pathways, such as NF-κB, Wnt/β-catenin, and STAT3, will pave the way for more effective and personalized treatments for colon cancer.

## Figures and Tables

**Table 1:** Overview of studies investigating the role of TNF-α in colon cancer.

Study	Study Design	Sample Size	TNFα Status	Findings and Associated Outcomes
Al Obeed et al., 2014 [14]	Retrospective cohort study	30 colorectal cancer patients	↑ TNF-α (mRNA and protein)	Elevated expression associated with advanced stages (Stage III/IV) and contribute to tumor progression and immune evasion
Li et al., 2011 [13]	Case–control study	180 colon cancer patients and 180 control subjects	↑ TNF-α in 308AA genotype	Patients with 308AA genotype were associated with ↑ TNF-alpha levels and a ↑ risk of colon cancer
Kaminska et al., 2005 [15]	Observational study	157 untreated colorectal cancer patients and 50 healthy volunteers	↑ sTNF RI in 69% of CRC patients	sTNF RI is strongly associated with tumor grade, invasion, and worse prognosis and is an independent prognostic marker for overall survival
Kemik et al., 2010 [16]	Observational study	126 colon cancer patients and 36 controls	↑ TNF-α	Elevated TNF-α levels were observed in cachectic patients and this elevation contributes to the development and severity of cancer cachexia
Babic et al., 2016 [17]	Prospective cohort study	544 CRC patients (225 males and 319 females)	↑ sTNFR2 (TNF-alpha activity)	Poor overall survival and ↓ colorectal cancer-specific survival as sTNFR2 is linked to TNF-α pathway activation
Chan et al., 2011 [18]	Case–control study	280 cases of colorectal cancer and 555 matched controls	↑ inflammatory markers	Inflammatory markers increase the risk of CRC and anti-inflammatory drugs reduce inflammation leading to decreased colorectal cancer risk, particularly in those with elevated inflammatory markers
Kapitanovic, 2014 [19]	Case–control study	91 patients with sporadic colon adenocarcinoma and 100 healthy controls	↑ TNFα gene/protein	TNFα gene and protein expression levels were significantly resulting in escalated tumor progression and tumor growth in colon adenocarcinoma
Zhao and Zhang, 2018 [20]	Experimental study	HCT-116 colon cancer cells treated with different TNF-α concentrations	↑ TNF-α at low concentration	↑ TROP-2 promotes cancer cell motility and invasion
Li et al., 2017 [21]	Tissue sample analysis study	108 human colon cancer tissue samples	↑ TNF-α in colon cancer tissues and cell lines	Blocking TNF-α with infliximab negates the effect of TNF-α-driven tumor-promoting inflammation, it enhances chemotherapy effectiveness
M. Grimm et al., 2011 [22]	In vitro study	104 patients	↑ TNF-α in 94% of CRC patients	Increased TNF-α levels correlated with more aggressive disease and lymph node metastases
Guo et al., 2023 [23]	In vitro study	54 CRC and 60 gastric cancer samples.	↑ TNF-α/TNFR2 signaling	TNF-α contributes to Tregs activation and blocking TNF-α improves the immunotherapy response

**Table 2:** Mechanistic overview of TNF-α–induced tumorigenic processes in CRC.

Study	TNF-α Effect	Mechanism/Pathway	Pro- or Anti-Tumorigenic	Human Models Used
Zhao & Zhang, 2018 [20]; Hamilton et al., 2011 [24]	Proliferation/Survival	NF-κB, STAT3 activation; TNFR2 upregulation	Pro-tumorigenic	Human colon cancer cell lines (e.g., HCT-116)
Zhao & Zhang, 2018 [20]	Migration/Invasion	TROP-2, MMP-9, ERK1/2, EMT, CXCL10/CXCR3 axis	Pro-tumorigenic	Human colon cancer cell lines, patient samples
Alotaibi et al., 2021 [25]	DNA damage	JNK pathway, especially with dietary carcinogens	Pro-tumorigenic	Human colorectal epithelial cell lines
Zhu et al., 2013 [26]	Stromal Interaction	TNFα-activated stromal COX-2 increases invasiveness	Pro-tumorigenic	Human stromal/epithelial co-culture models
Romagny et al., 2018 [27]	Switch to cell death	S-nitrosylation of cIAP1 alters TNFR1 signaling to apoptosis	Anti-tumorigenic	Human colon cancer cells
Nair et al., 2023 [28]	Contextual sensitization	Inhibition of RNA Pol III enhances TNFα-mediated cell death	Anti-tumorigenic	Human colon cancer cells (e.g., HCT-116)

**Table 3:** Therapeutic targeting of TNF-α in colon cancer: Mechanisms, monoclonal antibody therapy, and clinical implications.

Aspect	Details
Biological Role of TNF-α	Plays a critical role in colon cancer progression. High expression of TNF-α correlates with poor prognosis [21,49].
Mechanisms of Action	Stimulates macrophages to produce pro-tumorigenic factors such as CSF-1, VEGF-A, and MMP-2 [50]. Enhances epithelial-to-mesenchymal transition (EMT), increasing invasiveness [51].
Therapeutic Targeting	TNF-α is a key target in colon cancer due to its involvement in tumor-stromal interactions and inflammatory pathways.
Monoclonal Antibody Therapy	Infliximab (anti-TNF-α monoclonal antibody) induces antibody-dependent cellular cytotoxicity (ADCC) and complement-dependent cytotoxicity (CDC), promoting tumor cell apoptosis [56].
Combination Therapy	Synergistic effects when combined with chemotherapy agents like oxaliplatin or 5-fluorouracil, resulting in tumor regression in preclinical models [21,56].

## Data Availability

Not applicable.

## Abbreviations

The following abbreviations are used in this manuscript:

TNF-α	Tumor necrosis factor alpha
NF-κB	Nuclear factor kappa-light-chain-enhancer of activated B cells
STAT3	Signal transducer and activator of transcription 3
CRC	Colorectal cancer
DNA	Deoxyribonucleic acid
ROS	Reactive oxygen species
Wnt	Wingless-type
EMT	Epithelial mesenchymal transition
TNFR1	TNF-α type 1 receptor
TNFR2	TNF-α type 2 receptor
HT-29	Human colorectal adenocarcinoma cell line
IBD	Inflammatory bowel disease
IKK	IκB kinase
TRAF2	Tumor necrosis factor receptor-associated factor 2
RIP1	Kinase receptor interacting protein 1
NEMO	Nuclear factor-kappa B essential modulator
8-OHdG	8-hydroxydeoxyguanosine
GG-NER	Global Genome NER
DNMTs	DNA methyltransferases
TROP-2	Tumor-associated calcium signal transducer 2
